# Reduced frontopolar brain activation characterizes concussed athletes with balance deficits

**DOI:** 10.1016/j.nicl.2020.102164

**Published:** 2020-01-11

**Authors:** I. Helmich, J. Coenen, S. Henckert, E. Pardalis, S. Schupp, H. Lausberg

**Affiliations:** a Department of Neurology, Psychosomatic Medicine and Psychiatry, Institute of Health Promotion and Clinical Movement Science, German Sport University (GSU) Cologne, Am Sportpark Müngersdorf 6, 50933 Cologne, Germany; b Department of Sport and Health, Institute of Sport Medicine, Paderborn University, Warburger Str. 100, 33098 Paderborn, Germany

**Keywords:** Sport-related concussions, fNIRS, Postural control, Brain oxygenation, Frontopolar cortex

## Abstract

**Objectives:**

Athletes with sport-related concussions (SRC) often demonstrate deficits in postural stability. Lower cerebral blood flow in frontal cortices has been documented in athletes with symptoms after SRC, however, it is unclear if functional brain oxygenation during postural control tasks is reduced in symptomatic athletes after SRC in the same manner. We therefore compared brain oxygenation patterns in frontal cortices of symptomatic and asymptomatic athletes with SRC during postural control tasks with the hypothesis that symptomatic athletes are characterized by reduced functional brain oxygenation during postural control.

**Methods:**

62 concussed athletes (*n* = 31 symptomatic, *n* = 31 asymptomatic) were investigated during four postural control tasks with eyes closed versus eyes opened conditions and stable vs. unstable surface conditions. Brain oxygenation was assessed using functional NearInfraRed Spectroscopy (fNIRS) on frontopolar cortices of each hemisphere. Postural sway was measured by the analysis of ground reaction forces.

**Results:**

Symptomatic athletes showed greater postural sway when compared to asymptomatic athletes during postural control, particularly during closed eyes and/or unstable surface conditions. Changes of oxygenated hemoglobin (∆HbO_2_) within the left hemispheric frontopolar cortex were significantly reduced in symptomatic athletes when compared to asymptomatic athletes during the eyes closed condition. A stepwise linear regression analysis revealed that self-reported post-concussion symptoms such as headaches and sadness predict decreased brain oxygenation during postural control with closed eyes.

**Conclusion:**

Symptomatic athletes with increased postural sway are characterized by decreased frontopolar brain oxygenation during postural control tasks, particularly during conditions with closed eyes. Because the frontopolar cortex showed to be involved in redistributing executive functions to novel task situations, we conclude that athletes with post-concussion symptoms suffer from a deficit in coordinating postural adjustments to balance control tasks with reduced sensory input.

## Highlights

• Symptomatic athletes with balance deficits present reduced frontopolar oxygenation during postural control with closed eyes.
• Decreased brain oxygenation in the FPC of symptomatic individuals may characterize the deficit of shifting the focus from visual inputs towards proprioception.

## 1 Introduction

Although concussions (/mild Traumatic Brain Injuries mTBI) may never be completely eliminated from sports, improved understanding of post-concussion sequela on the health status is necessary to prevent athletes from long-term impairments. Potential post-concussion health deficits concern symptoms such as headaches, dizziness, memory problems, etc. that usually last for about a week (Guskiewicz et al., 2001). However, long-term neuropsychological (Deb et al., 1998), psychiatric (Finkbeiner et al., May) or physical impairments such as gait or posture control (Howell et al., 2017; Ingersoll and Armstrong, 1992) have been also reported after sport-related concussions (SRC).

Broglio and Puetz (2008) pointed out that there is a lack of published studies on postural control after concussions. The assumption that balance decrements resolve within three to five days post-injury (Guskiewicz, 2003; McCrea et al., 2003) is contrasted by studies that report longer recovery times when more sensitive measurement devices are being used (Broglio and Puetz, 2008; Ingersoll and Armstrong, 1992; Thompson et al., 4). In fact, Thompson et al. (2005) measured postural instability in concussed subjects about three months past the incident. Ingersoll and Armstrong (Ingersoll and Armstrong, 1992) reported a greater distance of the center of pressure of individuals with fewer postural corrections more than one year post-concussion. Thus, the application of more sensitive measures of postural stability indicates that alterations of postural control after mTBI in sports might present as a long-term impairment.

Guskiewicz et al. (1997) reported that concussed athletes may suffer from sensory interaction problems as they demonstrated decreased stability during closed eyes conditions without any neuropsychological deficits. The authors hypothesized that mTBI athletes are unable to respond to altered environmental conditions and therefore select a motor response based on wrong cues (Guskiewicz et al., 1997). However, no group differences were evident during tilted support surface conditions (Guskiewicz et al., 1997). Thus, it remains unclear whether concussions represent a sensory interaction problem that is related to visual or tactile alterations. Further analyses showed that concussed athletes demonstrate postural control deficits during altered visual conditions (closed eyes) and during the combination of altered visual and sensory (tilted support surface) conditions indicating that concussed athletes are not using information from the vestibular and visual systems effectively (Guskiewicz, 2003). However, studies that actually provide data about the neuronal correlates of concussed athletes during postural control performances are scarce (Helmich et al., 2016; Thompson et al., 2005).

In concussed athletes who were already cleared for sport participation, the application of electroencephalography (EEG) revealed a decrease in EEG power in all bandwidths especially during standing postures (Thompson et al., 2005). The combination of postural control measurements (force plate system) with functional NearInfraRed Spectroscopy (fNIRS) imaging provided evidence that concussed individuals with persisting symptoms are characterized by decreased brain oxygenation patterns in frontal cortices when compared to a healthy control group during balance control (Helmich et al., 2016). However, increased brain oxygenation patterns during the combinational alteration of visual (eyes closed) and tactile (unstable surface) manipulations were also observed in frontopolar cortices of concussed individuals with persisting post-concussion symptoms and when compared to asymptomatic athletes with mTBI and non-concussed controls (Helmich et al., 2016). In fact, several studies reported contrasting results of increased (Helmich et al., 2016; McAllister et al., 2001) as well as decreased functional brain activation in frontal cortices (Chen et al., 2007; Helmich et al., 2015) of concussed individuals, particularly in the frontopolar cortex (FPC) (Helmich et al., 2016; Chen et al., 2007; Helmich et al., 2015). Thus, it remains unclear whether concussed athletes are characterized by functional hyper- or hypoactivity in the frontal cortex during postural control tasks. Resting-state analyses using EEG showed that athletes with sport-related concussions are characterized by decreased activity in the FPC that is additionally negatively correlated to post-concussion symptoms (Virji-Babul et al., 2014). Athletes reporting greater symptoms also showed lower frontal cerebral blood flow following acute concussion (Churchill et al., 2017). Because fNIRS showed to be a valid tool to investigate brain oxygenation patterns during postural control tasks (Basso Moro et al., 2014; Beurskens et al., 2014; Ferrari et al., 2014; Fujimoto et al., 2014; Fujita et al., 2016; Helmich et al., 2016; Herold et al., 2017; Huppert et al., 2013; Karim et al., 2013; Karim et al., 2012; Lin et al., 2017; Mahoney et al., 2016; Mihara et al., 2008; Takakura et al., 2015; Wang et al., 2016), particularly in the frontal cortex as this area is modulated by task difficulty during postural control (Basso Moro et al., 2014; Eckner et al., 2011; Guskiewicz et al., 1997; Howell et al., 2017), we investigate in the present study the hypothesis that athletes with mTBI and post-concussion symptoms show decreased brain oxygenation patterns in the FPC during postural control tasks that are characterized by reduced sensory information such as when balancing with closed eyes.

## 2 Materials and methods

### 2.1 Participants

62 active athletes with a history of SRC (mean age: 25.7 ± 5.3 years; 22 female, 40 male; average years of sports participation: 8.7 ± 6.6) from various sports (American Football, ice hockey, rugby, boxing, handball, soccer, etc.) from local sports-clubs participated in the study as part of a concussion assessment protocol of the German Sports University. Written informed consent was obtained from each participant. The local Ethics Committee of the GSU approved the study.

### 2.2 Clinical assessment

Participants were clinically questioned using a standardized questionnaire to obtain the athletes’ sports participation, age, education, and the occurrence of a mild traumatic brain injury as defined by the recent consensus statement on concussion in sport (McCrory et al., 2013), time post-concussion, and the presence or absence of post-concussive symptoms according to symptom scale used in the “Sport Concussion Assessment Tool – 3rd edition” (SCAT3) (Guskiewicz et al., 2013). The number of 22 symptoms was summated to a post-concussion symptom score (PCS score), with a maximum of 132 (22 × 6). We used a PCS score of 10 as a cut-off to differentiate between symptomatic and asymptomatic athletes as previous studies reported an average between 8 and 10 points during baseline tests (Chen et al., 2007; Lovell et al., 2004) (Table 1). Asymptomatic athletes were matched to symptomatic athletes in age, gender, amount of experienced SRC, time post-concussion, years of sports participation, and cognitive performance (working memory). I.e., there were no significant group differences in chi-square tests and independent t-tests.

**Table 1:** Participants (*significant differences between groups).

	Asymptomatic athletes	Symptomatic athletes
Number of participants	31	31
Gender (female/male)	9/22	13/18
Age	24.4 ± 4.0	26.9 ± 6.2
PCS score*	0.9 ± 0.9	27.1 ± 14.9
Experienced concussions	2.3 ± 2.6	2.5 ± 1.8
Time post-concussion (months)	51.1 ± 56.0	27.9 ± 47.5
Years of sport participation	7.4 ± 4.4	10.0 ± 8.0
Working memory performance (correct answers in%)	89 ± 0.0	86 ± 0.1
Response times during working memory performance (milliseconds)*	981.8 ± 145.9	1071.7 ± 162.8

### 2.3 Neuropsychological testing

Participants performed a working memory task, which had proven effective in the investigation of functional abnormalities of concussed athletes (Chen et al., 2004; Helmich et al., 2015). During the working memory task, four out of five items were presented in random order at the center of a computer screen. After the presentation of the fourth item, a delay of 1 s was introduced. Immediately after this delay, a test item was presented, and the subject had to indicate whether this test item was one of the four items presented before the delay or if the item had not been presented. The subjects indicated their responses by pressing a mouse button (right button = yes, left button = no). Each subject had 1.5 s to respond, after which a new trial began. The participants had the opportunity to practice the task before in order to familiarize themselves with the target stimuli. Variables of the working memory task for statistical analysis constituted the correct answers (%) and the response times in milliseconds (Table 1).

### 2.4 Posturography, balance tasks and data collection

**Fig. 1:**
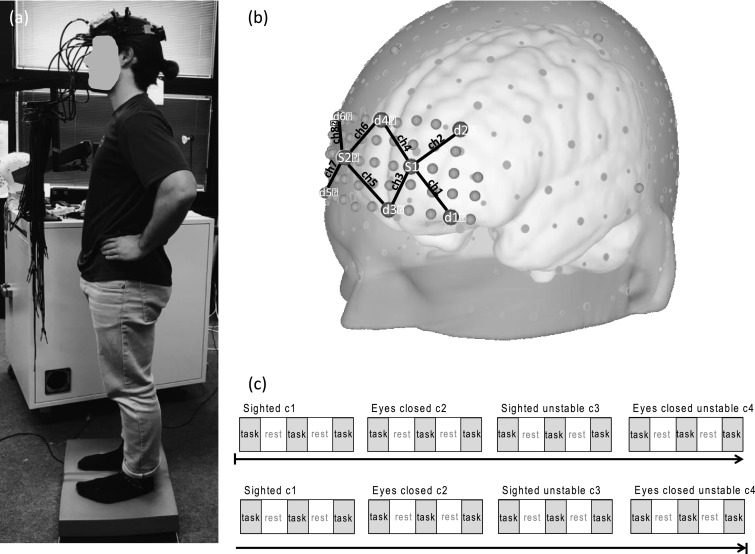
Left (a): Balance position of one individual during the *unstable* surface condition; top right (b): fNIRS optode placement (s1-s2: sources; d1-d6: detectors; ch1-ch8: channels) above the frontopolar cortex of the right (RH) and left hemispheres (LH) according to the 10–20-system; bottom right (c): Experimental conditions and block design.

Four balance conditions (six trials per condition (two blocks with three trials each; 10 s per trial; Fig. 1) were carried out according to Shumway-Cook and Horak (Shumway-Cook and Horak, 1986), which examine combinations of visual and tactile manipulations during balance control: condition 1: *eyes opened* (c1); condition 2: *eyes closed* (c2). The two conditions (c1 and c2) were performed either on a firm (/*stable*) surface or on an *unstable surface*: condition 3 (c3): *eyes opened* and *unstable surface*; condition 4 (c4): *eyes closed* and *unstable surface*. The *unstable* surface was created using a piece of six cm thick foam pad ("AIREX Balance-Pad“). Each balance condition comprised two blocks, each of which included three trials (ten seconds per trial), resulting in a total of six trials per condition. The subjects were instructed to stand still on both feet (distance between feet: 2 cm) without losing balance in a standardized position and posture (Fig. 1).

During balance tasks, a force plate system („ZEBRIS platform, type FDM-S”, measure frequency 240 Hz) was used to register center of mass displacement (/postural sway) by measuring ground reaction forces. This system provides three parameters of information about the ability to keep postural control, i.e., (i) it registers the deviations from the Center of Pressure (CoP) by the mean length of the movement path per time [millimeters/second] (path length, PL); PL is defined as the absolute length of the CoP path movements throughout the testing period; (ii) the second parameter surface area (SuA) [mm^2^] is defined as a 95% confidence ellipse for the mean of the CoP anterior, posterior, medial and lateral coordinates; (iii) the third parameter velocity (V) [mm/second] represents the mean velocity during the postural control trials per second. Mean parameters of the values of PL, SuA, and V were exported for each subject and condition for statistical analyses.

### 2.5 fNIRS acquisition and analysis

Cerebral oxygenation changes were recorded during postural control tasks using a near-infrared optical tomographic imaging device (DYNOT Imaging System, NIRx, Wavelengths 760 nm, 830 nm, Sampling rate 7.2 Hz). Methodology and underlying physiology are explained in detail elsewhere (Cope et al., 1988; Obrig and Villringer, 2003). A total of 8 optodes (2 emittors, 6 detectors) were placed above the frontopolar cortex (FPC) of each hemisphere resulting in 8 channels of measurement (channel 1–4: FPC of the left hemisphere (LH); channel 5–8: FPC of the right hemisphere (RH); Fig. 1). Optodes were placed with an approximate interoptode distance of 3 cm according to the 10–20-system (Jasper, 1958). Optodes were mounted with a customized plastic hard shell system on the participant's head to gain placement stability and to avoid movement artifacts.

Data were analyzed using the “nirslab” software package (NIRx Medical Technologies, LLC). 8 channels (ch) were converted to hemoglobin concentration changes according to Cope et al. (1988). The „remove discontinuities “ and the „remove spike artifacts “ algorithms of the nirsLAB toolbox were used to correct for discontinuities and spike artifacts in the (raw) signal (with the standard deviation threshold set to 5). When removing spike artifacts, data was replaced by using the “nearest signals“ function. Data were then bandpass filtered (low cut-off frequency at 0.01 Hz / high cut-off frequency at 0.2 Hz) to eliminate the effects of heartbeat, respiration, and low frequency signal drifts for each wavelength. Because individuals were asked to stand still during the entire procedure, the baseline was set to the full time course of the data set. Block averages (10 s) of ∆HbO2 from each channel and condition were then exported for statistical analyses.

### 2.6 Statistics

Comparisons of the mean(s) (repeated (rmANOVA) and univariate (uniANOVA) analyses of variance) were performed using IBM SPSS statistics (Version 25). The parameters path length (PL), parameter surface area (SuA), and velocity (V) were used for statistical analyses of postural control. Statistical analyses of brain oxygenation data focused on the changes of oxygenated hemoglobin (∆HbO_2_), because these appear to reflect task-related cortical activation more directly than changes of deoxygenated hemoglobin, as evidenced by the stronger correlation between the former and the blood-oxygenation level- dependent signal measured by fMRI (Strangman et al., 2002) and by the results of animal studies (Hoshi et al., 1985). Thus, mean brain oxygenation patterns (block averages of 10 s) of ∆HbO_2_ were used for statistical analyses of brain activity. The between-subjects factor *group* constitutes (i) concussed athletes with a PCS score > 10 (*symptomatic*), and (ii) concussed athletes with a PCS score < 10 (*asymptomatic*). Repeated within-subjects factors constitute *visibility* (postural control conditions (i) with either opened eyes or (ii) closed eyes) and *stability* (postural control conditions while standing (i) on a stable or (ii) on an unstable surface). For fNIRS analyses, the additional within-subjects factor *channels* (8) was statistically calculated by uniANOVA. Significant results are reported from *p* < 0.05. Multiple post hoc pairwise comparisons were corrected with Bonferroni corrections. To determine a relationship of postural control, brain oxygenation, the PCS score, and post-concussion symptoms, we calculated a correlation (using the Pearson's correlation coefficient, r_p_) and (stepwise) regression analyses. Because the aim of the present paper is to better understand differences between symptomatic and asymptomatic athletes, we focus onto the effects between groups in the results section.

## 3 Results

### 3.1 Group effects

#### 2.1.1 Participants

Significant differences between groups were found for the PCS score (t(60) = −9.703, *p* < 0.001) and the response times during working memory performances (t(60) = −2.289, *p* < 0.05; Table 1). Symptomatic athletes (Mean [M] = 27.1 ± 14.9) showed significantly higher PCS scores than asymptomatic athletes (*M* = 0.9 ± 0.9). Furthermore, symptomatic athletes (*M* = 1071.7 ± 162.8) showed significantly increased response times during the working memory task when compared to asymptomatic athletes (*M* = 981.8 ± 145.9).

#### 2.1.2 Balance performance

The rmANOVA revealed for the parameter surface area (SuA) (but neither for path length nor velocity) significant differences between *groups* (F(1, 60) = 7.874, *p* < 0.01, η^2^ = 0.116), an interaction effect of *group* x *visibility* (F(1, 60) = 7.093, *p* < 0.05, η^2^ = 0.106), *group* x *stability* (F(1, 60) = 6.646, *p* < 0.05, η^2^ = 0.100), and of *group* x *visibility* x *stability* (F(1, 60) = 5.096, *p* < 0.05, η^2^ = 0.078; Table 2).

**Table 2:** Overview of the (significant) results (mean ± standard error) between groups.

Postural control	Asymptomatic athletes	Symptomatic athletes
SuA	497.4 ± 61.6	741.9 ± 61.6
SuA sighted	164.8 ± 27.6	248.7 ± 27.6
SuA eyes closed	829.9 ± 102.3	1235.1 ± 102.3
SuA stable	82.6 ± 14.4	141.4 ± 14,4
SuA unstable	912.1 ± 112.1	1342.4 ± 112.1
SuA eyes closed / stable	276.6 ± 48.6	423.1 ± 48.6
SuA sighted / unstable	112.2 ± 21.9	208.5 ± 21.9
SuA (mm^2^) eyes closed / unstable	1547.6 ± 189.4	2261.6 ± 189.4

Brain oxygenation	Asymptomatic athletes	Symptomatic athletes
∆HbO_2_ (ch1: LH FPC)	0.000016 ± 0.000052	−0.000058 ± 0.000052
∆HbO_2_ (ch4: LH FPC)	0.000147 ± 0.000043	0.000018 ± 0.000043
∆HbO_2_ (ch6: RH FPC)	0.000029 ± 0.000043	−0.000031 ± 0.000043

Values presented are surface area, SuA (mm^2^); brain oxygenation (changes of oxygenated hemoglobin, ∆HbO_2_) within channels (ch) 1,4, and 6; left hemisphere, LH; frontopolar cortex, FPC; right hemisphere, RH.

**Fig. 2:**
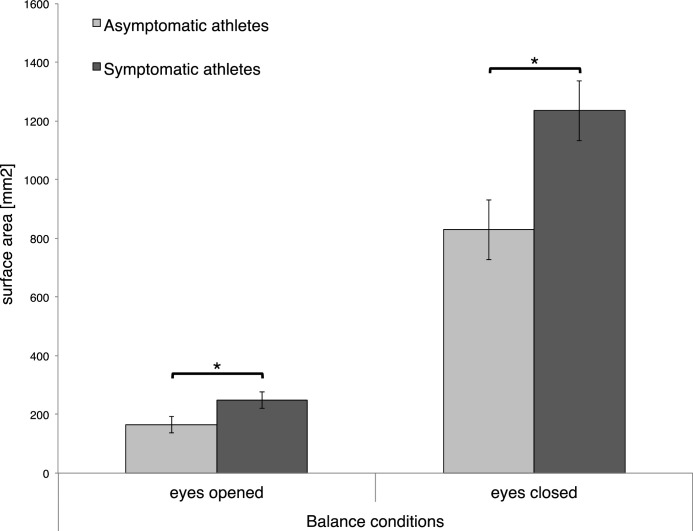
Mean surface areas (mm^2^) of *symptomatic* and *asymptomatic* athletes during balance conditions with *eyes opened* vs. *eyes closed*.

Post-hoc comparisons of the *group* effect revealed a significantly greater SuA for *symptomatic* athletes when compared to *asymptomatic* (*p* < 0.01). Post-hoc comparisons of the interaction effect of *group* x *visibility* revealed significantly greater SuA for *symptomatic* athletes when compared to *asymptomatic* during the *sighted* (*p* < 0.01) as well as during the condition with *closed eyes* (*p* < 0.01; Fig. 2). Post-hoc comparisons of the interaction of *group* x *stability* revealed significantly greater SuA for *symptomatic* athletes when compared to *asymptomatic* during the *stable* (*p* < 0.01) as well as during the *unstable* conditions (*p* < 0.01). Post-hoc comparisons of the interaction of *group x stability* x *visibility* revealed significantly greater SuA for *symptomatic* athletes when compared to *asymptomatic* during the *stable* condition with *closed eyes* (*p* < 0.01), and during the *unstable* condition with *opened* (*p* < 0.05) and *closed eyes* (*p* < 0.01).

#### 2.1.3 Correlation of balance performance and the PCS score

**Fig. 3:**
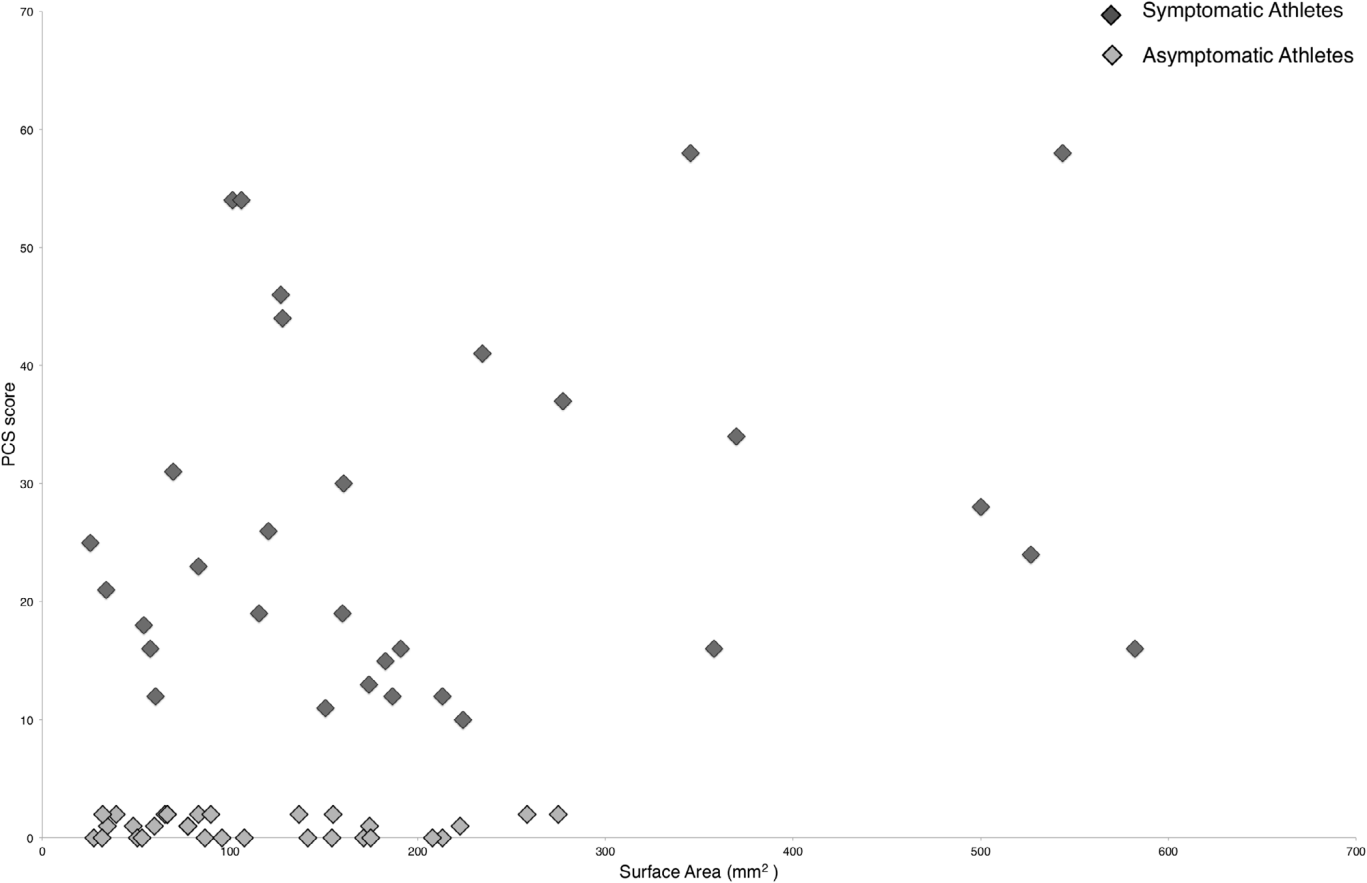
Correlation of the postural control (note: increased mean surface areas (mm^2^) indicate decreased postural control) and the PCS scores of *symptomatic* and *asymptomatic* athletes during balance conditions with *eyes closed*.

There is a significant positive correlation between the (increased) PCS score and worse postural control by increased mean surface areas during the *eyes opened* and *stable surface* condition (r_s_(62) = 0.306, *p* < 0.05), during the *eyes closed* and *stable surface* condition (r_s_(62) = 0.376, *p* < 0.01), during the *eyes opened* and *unstable surface* condition (r_s_(62) = 0.282, *p* < 0.05), and during the *eyes closed* and *unstable surface* condition (r_s_(62) = 0.275, *p* < 0.05). A following stepwise linear regression analysis with the PCS score as the dependent variable and the significantly correlated SuA parameters as independent variables revealed significance (F(1, 60) = 9.893, *p* < 0.01, *R*^2^ = 0.142), i.e., the SuA during the *eyes closed* and *stable surface* condition significantly predicted the PCS score (β = 0.376, *t* = 3.145, *p* < 0.01; Fig. 3).

#### 2.1.4 Correlation of balance performance and response times

It exists a significant positive correlation between (increased) mean surface area during the *eyes closed* condition and increased) response times during the working memory task (r_s_(62) = 0.279, *p* < 0.05).

#### 2.1.5 Brain oxygenation

**Fig. 4:**
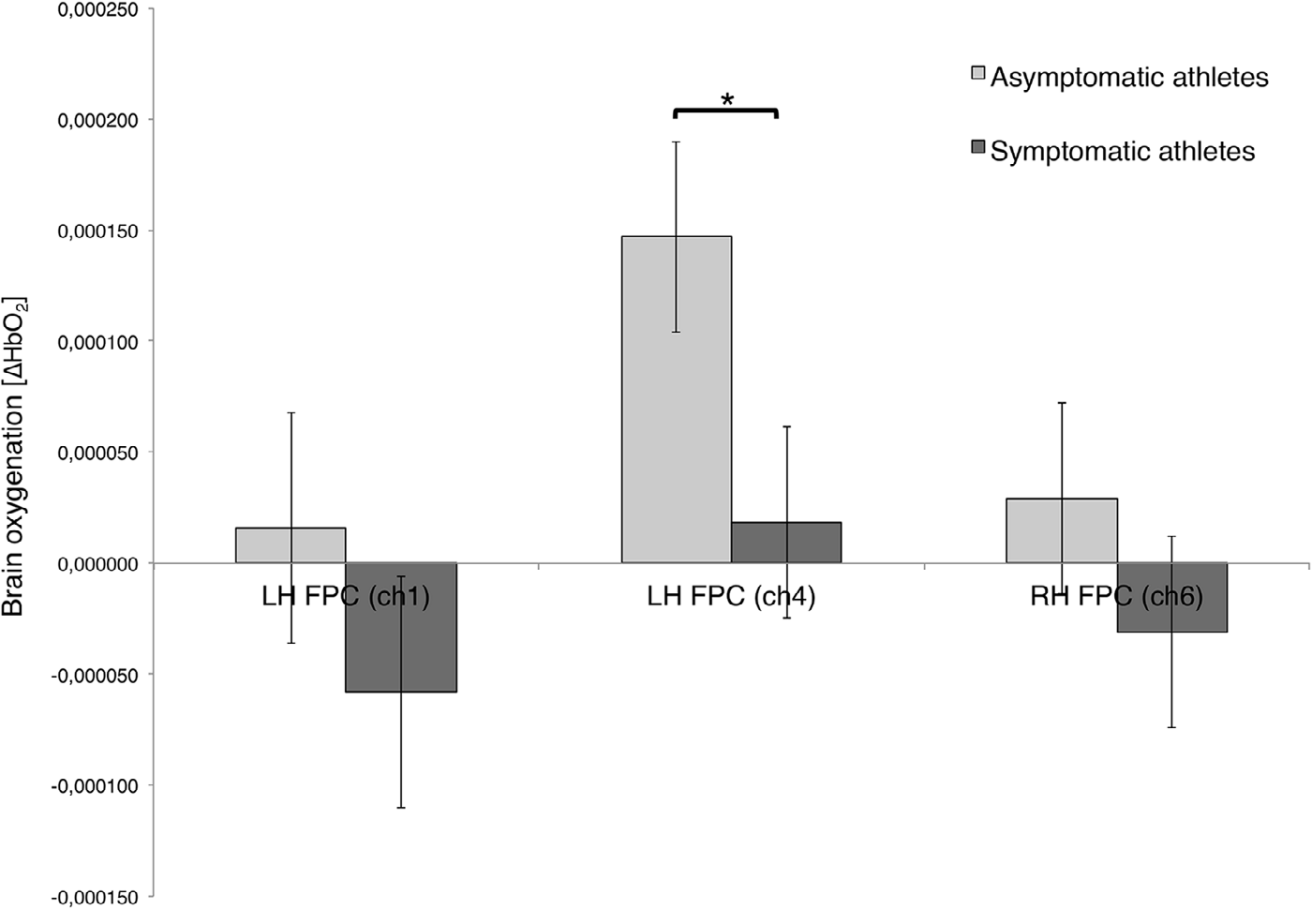
Mean brain oxygenation (∆HbO_2_) in the left hemispheric (LH) frontopolar cortex (FPC, channel 1 and channel 4), and in the FPC of the right hemisphere (channel 6) between *symptomatic* and *asymptomatic* groups during balance conditions with *eyes closed*.

The rmANOVA revealed a significant effect for the interaction of *group* x *visibility* (F(8, 53) = 3.071, *p* < 0.05, η^2^ = 0.317). The uniANOVA showed significant effects above the right and left hemispheric (LH) frontopolar cortex (FPC) for the interaction of *group* x *visibility* for channel 1 (ch1; LH FPC; F(1, 60) = 4.799, *p* < 0.05, η^2^ = 0.074), ch4 (LH FPC; F(1, 60) = 7.215, *p* < 0.05, η^2^ = 0.107; Fig. 4), and channel 6 (RH FPC; marginally significant, F(1, 60) = 3.394, *p* = 0.07, η^2^ = 0.05; table 2). Post-hoc comparisons revealed reduced ∆HbO_2_ in *symptomatic* when compared to *asymptomatic* athletes during the condition with *closed eyes* in all three channels, however, only in channel 4 post-hoc comparisons reached significance (*p* < 0.05).

#### 2.1.6 Correlation of brain oxygenation and PCS score

**Fig. 5:**
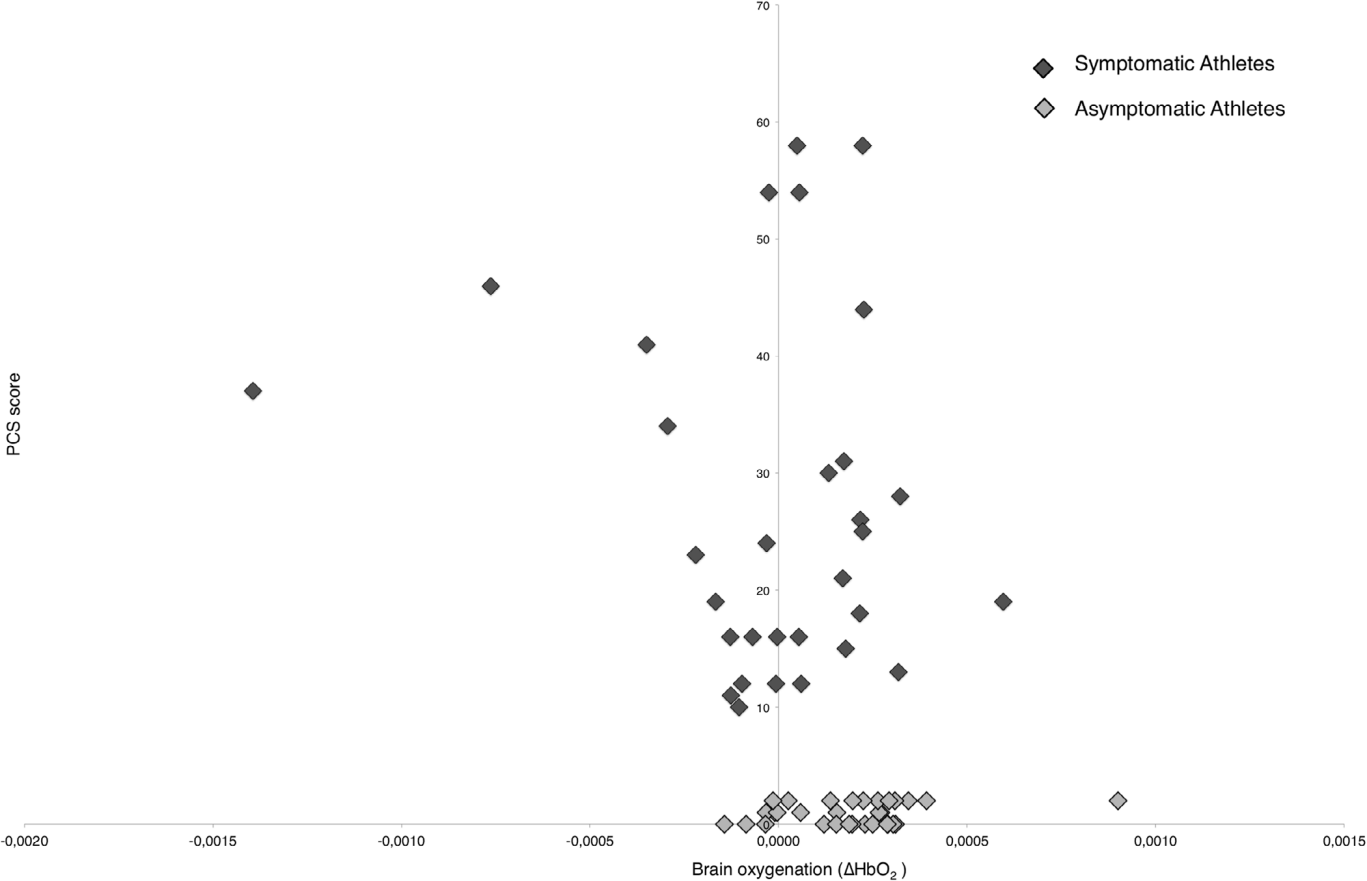
Correlation of the PCS score and brain oxygenation in channel 4 (∆HbO_2_) during the *eyes closed* and *stable surface* condition.

It exists a significant negative correlation between the (increased) PCS score and the (decreased) brain oxygenation during the *eyes closed* condition in channel 4 (r_s_(62) = −0.345, *p* < 0.01; Fig. 5). A following stepwise linear regression analysis with the PCS score as the dependent variable and all fNIRS channels as independent variables revealed significance (F(1, 60) = 8.572, *p* < 0.01, *R*^2^ = 0.125), i.e., left hemispheric FPC (channel 4) during the *eyes closed* and *stable surface* condition significantly predicted the PCS score (β = −0.354, *t* = −2.928, *p* < 0.01).

#### 2.1.7 Correlation of brain oxygenation and post-concussion symptoms

A following correlation analysis of the ∆HbO_2_ in channel 4 during the *eyes closed* and *stable surface* condition and each post-concussion symptom revealed significance, i.e., the data showed a significant negative correlation between (decreased) ∆HbO_2_ in the LF FPC (ch4) and the (increased) symptoms *headaches* (r_s_(62) = −0.461, *p* < 0.001), *pressure in the head* (r_s_(62) = −0.276, *p* < 0.05), *sensitivity to light* (r_s_(62) = −0.316, *p* < 0.05), *sensitivity to noise* (r_s_(62) = −0.329, *p* < 0.01), *difficulty remembering* (r_s_(62) = −0.309, *p* < 0.05), *fatigue or low energy* (r_s_(62) = −0.267, *p* < 0.05), *confusion* (r_s_(62) = −0.276, *p* < 0.05), *difficulty falling asleep* (r_s_(62) = −0.376, *p* < 0.01), *irritability* (r_s_(62) = −0.352, *p* < 0.01), and *sadness* (r_s_(62) = −0.347, *p* < 0.01). A following stepwise linear regression analysis with ∆HbO_2_ in ch4 during the *eyes closed* and *stable surface* condition as the dependent variable and the significantly correlated symptoms as independent variables revealed significance (F(2, 59) = 12.327, *p* < 0.001, *R*^2^ = 0.295), i.e., the (increased) symptoms *headaches* (β = −0.421, *t* = −3.819, *p* < 0.001) and *sadness* (β = −0.290, *t* = −2.627, *p* < 0.05) significantly predicted decreased ∆HbO_2_ in LF FPC (ch4) during the *eyes closed* and *stable surface* condition.

## 4 Discussion

The present study compared postural control performances in concussed athletes with and without post-concussion symptoms by analyzing postural sway and functional brain oxygenation in frontopolar cortices using fNIRS. Symptomatic athletes presented increased postural sway (/surface areas (SuA)) when compared to asymptomatic athletes overall balance conditions as well as during the *eyes closed* condition, the *unstable surface* condition, and the combination of *closed eyes* and *unstable surface* condition. The SuA during the *eyes closed* and *stable surface* condition showed to predict the PCS score. The analysis of the fNIRS data revealed that symptomatic athletes are characterized by a lack of activation (i.e., reduced changes of ∆HbO_2_) in frontopolar cortices when compared to asymptomatic athletes during postural control with eyes closed on a stable surface. The symptoms *headaches* and *sadness* significantly predicted reduced ∆HbO_2_ in frontopolar cortices when controlling posture with *closed eyes*.

### 4.1 Postural control

In line with previous findings (Kleffelgaard et al., 2012; Purkayastha et al., 2019; Schmidt et al., 2018), the present study showed that symptomatic athletes present balance deficits when compared to asymptomatic athletes, particularly during conditions with eyes closed. Guskiewicz et al. (1997) pointed out that concussed athletes may suffer from sensory integration problems. Because concussions have been associated to a decline in the randomness of center of pressure oscillations (De Beaumont et al., 2011; Cavanaugh et al., 2005), it has been also assumed that the concussive injury constrains the output of the postural control system (Cavanaugh et al., 2006). Thus, the impaired control of balance with closed eyes of symptomatic athletes might be grounded in a deficit of integrating sensory input, particularly when visual information is missing, which then results in impaired motor output control.

The present analyses also revealed that symptomatic athletes respond slower (/increased response times) during a working memory task when compared to the asymptomatic group. Increased symptoms post concussion have been previously been associated to increased response times (Chen et al., 2007; Eckner et al., 2011). Furthermore, increased postural sway during eyes closed conditions and increased response times during working memory performances are positively correlated. Data from studies about neurodegenerative disorders also showed a relationship of reduced response times (during an auditory stepping task) and postural control deficits (VanderVelde et al., 2005). Because neurodegeneration following repetitive concussions has been related to motor deficits and cognitive dysfunction (Baugh et al., 2012), the present data indicates that symptomatic athletes might be particularly impaired in the time to adapt to altered sensory manipulations during postural control tasks. Further investigations must therefore differentiate whether alterations of postural control are particularly related to decreased reaction times.

The analysis of the fNIRS data revealed that symptomatic athletes are characterized by a lack of activation (i.e., reduced changes of ∆HbO_2_ when compared to asymptomatic athletes) in frontopolar cortices when performing postural control tasks with closed eyes. When balancing with closed eyes, the attention of an individual shifts from external reference points towards the perception of proprioceptive information from the own body (El Shemy, 2018 Dec). Marx et al. (2003) postulated that during eyes closed conditions, the mental activity of an individual shifts from an “exteroceptive” state during eyes opened conditions to an “interoceptive” state that is characterized by imagination and multisensory activity that also depends on information from frontopolar cortices. Thus, individuals must adapt to a novel situation and control posture based on altered proprioceptive inputs. As it has been postulated that concussed athletes may suffer from balance problems during situations with altered sensory inputs (Guskiewicz et al., 1997), the reduced brain oxygenation in the FPC of individuals may characterize the deficit of shifting the focus from visual inputs towards proprioception. The FPC contributes to the exploration and rapid acquisition of novel behavioral options, which constitutes an essential aspect of complex, higher order behavior (Boschin et al., 2015). FPC-lesioned monkeys remained more focused than control monkeys in exploiting a current task than when they faced newly introduced interruptions by a secondary task suggesting that the FPC is involved in redistribution of cognitive resources from the current task to novel situations (Mansouri et al., 2015). Furthermore, the prefrontal cortex showed to be involved in active controlled processing for the disambiguation of vibrotactile information in short-term memory (Kostopoulos et al., 2007). Thus, the FPC seems to critically contribute to posture control when integrating (proprioceptive) information during altered sensory inputs. The decreased oxygenation of symptomatic athletes therefore indicates that those individuals suffer from the inability to adapt to postural control conditions with altered sensory inputs such as balance conditions with closed eyes.

### 4.2 Post-concussion symptoms and postural control

Further analyses revealed that increased self-reported symptoms such as *headaches* and *sadness* predicted decreased brain oxygenation patterns in frontopolar regions during postural control conditions with closed eyes. Symptoms of *fatigue or low energy* have been reported previously to be related to balance deficits (Kitaoka et al., 2004; Lundin et al., 1993). It has also been documented that postural control, headaches, and concussions are related by the fact that concussed athletes with post-traumatic headache experience greater declines in balance than concussed athletes without posttraumatic headache (Register-Mihalik et al., 2008). Individuals affected by chronic tension-type headache are characterized by increased body sway particularly during balance tasks with eyes closed (Giacomini et al., 2004). Thus, when athletes suffer from post-concussion symptoms such as headaches, the present data indicate that those athletes are characterized by decreased brain oxygenation when controlling posture with closed eyes. Abnormal somatosensory afferents arising from the muscle spindles, joint and pain receptors, or nerve roots of the cervical spine can contribute to cervicogenic headache (Biondi, 2005; Kristjansson and Treleaven, 2009) and vertigo or dizziness (Kristjansson and Treleaven, 2009). Because concussive injuries can result in abnormal proprioceptive feedback (Mallinson and Longridge, 1998 Nov; Rubin et al., 1995), it has been assumed that individuals with post-traumatic headache experience increased balance deficits because sensory inputs are disrupted (Register-Mihalik et al., 2008). In fact, concussed athletes may suffer from balance problems during conditions with altered sensory inputs (Guskiewicz et al., 1997). Thus, decreased frontopolar brain oxygenation during postural control tasks with closed eyes points out that symptomatic athletes are impaired in the integration of sensory information to control posture, particularly when suffering from post-concussion headaches. However, the fact that mTBI increases the likelihood of depression and post concussion syndrome (Lange et al., 2011; Vanderploeg et al., 2007), i.e., mental disorders that are also commonly accompanied by symptoms such as *sadness* and *headaches,* must be taken into account as depressive patients have been characterized by decreased brain oxygenation in the frontal cortex as well (Schecklmann et al., 2011). Thus, future studies must elaborate whether decreased oxygenation patterns in the frontal cortex characterize concussed athletes with long-term impairments or if this pattern of brain oxygenation is related to the progression of a depressive disorder.

### 4.3 Practical implications

Because sport-related concussions and potential long-term effects are a major concern in sports (McCrory et al., 2013), it is of relevance to understand post-concussion outcomes on health status of athletes in order to make decisions about the return-to-play and / or treatment strategies. Recent development of portable instruments (Scholkmann et al., 2014) allow to address the potential application of fNIRS immediately after concussive incidents on site of sport events. This offers the unique possibility to assess brain oxygenation immediately post-concussion for potential clinical diagnosis. However, because clinical decisions have to be made for each athlete individually, a variety of issues must be taken into account when using NIRS clinically (Greenberg et al., 2017). NIRS measurements concern light absorption of chromophores from a small segment of tissue within the path of emitted light and its sensors, i.e., the data provides merely information about localized regional brain oxygenation (Scholkmann et al., 2014). Secondly, alterations in intra- and extracranial contents may affect readings (Gagnon et al., 2014; Tachtsidis and Scholkmann, 2016), however, at this point it is unknown what clinical impact extracranial contamination has on the use of NIRS devices (Greenberg et al., 2017). Although the clinical implications of these apparent inaccuracies require further study, they suggest that the brain oxygenation measurements using fNIRS do not solely reflect brain activation alone. Extra cerebral confounders can be minimized by several approaches such as for example multi-distance optode measurements (Gagnon et al., 2014; Tachtsidis and Scholkmann, 2016) or particular experimental designs (Tachtsidis and Scholkmann, 2016). To minimize confounders in the present study, we applied a block design contrasting between experimental tasks of similar characteristics that advances statistical calculations (Tachtsidis and Scholkmann, 2016). In view of those issues, the present fNIRS data indicate that symptomatic athletes present a deficit of activating neural structures that are relevant to control posture during altered sensory input, particularly during closed eyes conditions. Thus, symptomatic athletes might be particularly impaired to adapt to postural control conditions that are characterized by altered sensory inputs. Post-concussion headaches seem to particularly predict whether an individual suffers from decreased brain oxygenation patterns. Medical personal should therefore be aware that athletes who suffer from headaches might have deficits of integrating sensory information that is necessary to control posture.

## Declaration of Competing Interest

The authors disclose no conflicts of interest.
